# Perspective on the Multiple Pathways to Changing Brain States

**DOI:** 10.3389/fnsys.2020.00023

**Published:** 2020-05-08

**Authors:** Malinda L. S. Tantirigama, Timothy Zolnik, Benjamin Judkewitz, Matthew E. Larkum, Robert N. S. Sachdev

**Affiliations:** Institut für Biologie, Neurocure Center for Excellence, Charité Universitätsmedizin Berlin & Humboldt Universität, Berlin, Germany

**Keywords:** pyramidal neuron, brain state, cortico-thalamocortical, neuromodulation, active behavior, respiration

## Abstract

In this review article, we highlight several disparate ideas that are linked to changes in brain state (i.e., sleep to arousal, Down to Up, synchronized to de-synchronized). In any discussion of the brain state, we propose that the cortical pyramidal neuron has a central position. EEG recordings, which typically assess brain state, predominantly reflect the activity of cortical pyramidal neurons. This means that the dominant rhythmic activity that characterizes a particular brain state ultimately has to manifest globally across the pyramidal neuron population. During state transitions, it is the long-range connectivity of these neurons that broadcast the resultant changes in activity to many subcortical targets. Structures like the thalamus, brainstem/hypothalamic neuromodulatory systems, and respiratory systems can also strongly influence brain state, and for many decades we have been uncovering bidirectional pathways that link these structures to state changes in the cerebral cortex. More recently, movement and active behaviors have emerged as powerful drivers of state changes. Each of these systems involve different circuits distributed across the brain. Yet, for a system-wide change in brain state, there must be a collaboration between these circuits that reflects and perhaps triggers the transition between brain states. As we expand our understanding of how brain state changes, our current challenge is to understand how these diverse sets of circuits and pathways interact to produce the changes observed in cortical pyramidal neurons.

## Introduction

“Brain state,” particularly as it relates to consciousness, is usually recorded *via* EEG electrodes that predominantly measure the rhythmic activity of the cerebral cortex. Nevertheless, many circuits distributed across the brain contribute to and are related to the transition in brain states, and in this sense, the rhythmic activity of the cortex is a function of its bidirectional interaction with a host of subcortical structures. We know that changes in alertness, attention, arousal, and consciousness are associated with specific changes in levels and patterns of neuronal activity throughout the brain (Steriade and McCarley, 2005; Harris and Thiele, 2011). Explaining why and how the brain is in a given state at any given time is a formidable task. Like blind men considering the elephant (Ireland, 2007), this task has traditionally been approached from very different perspectives and has left the literature fragmented. A popular view is that system-wide transitions in brain state arise from ascending neuromodulatory input from the reticular activating system (reviewed in, Steriade and McCarley, 2005). Here, we offer a different perspective. We bring together several disparate ideas and discuss: (1) specific and non-specific thalamic nuclei; (2) neuromodulation and hypothalamus; (3) behavior; and (4) respiration as paradigms for explaining brain state transitions. Although each paradigm involves very different systems, weaving them together are the cortical pyramidal neurons. We propose that both the effect of input to the cortical pyramidal neurons and the effect of their output, especially the layer 5 pyramidal neurons, are key to understanding system-wide brain state transitions.

## Cortex, Thalamus and the Loop That Link Them to Brain States

There are two overriding reasons to focus on pyramidal neurons. Firstly, the brain state as detected by the EEG signal is almost entirely determined by the electrical field dipoles across the apical dendritic axis of pyramidal neurons (Buzsáki et al., 2012; Larkum, 2013; Suzuki and Larkum, 2017). But even more importantly, pyramidal neurons make up the majority (80%) of all cortical neurons and are the principal long-range cortico-cortically and subcortically projecting neurons in the brain (DeFelipe and Fariñas, 1992; Molyneaux et al., 2007). Their aggregate activity is therefore central to both the local manifestation of the brain state and the causal explanation for system-wide transitions between brain states.

**Figure 1:**
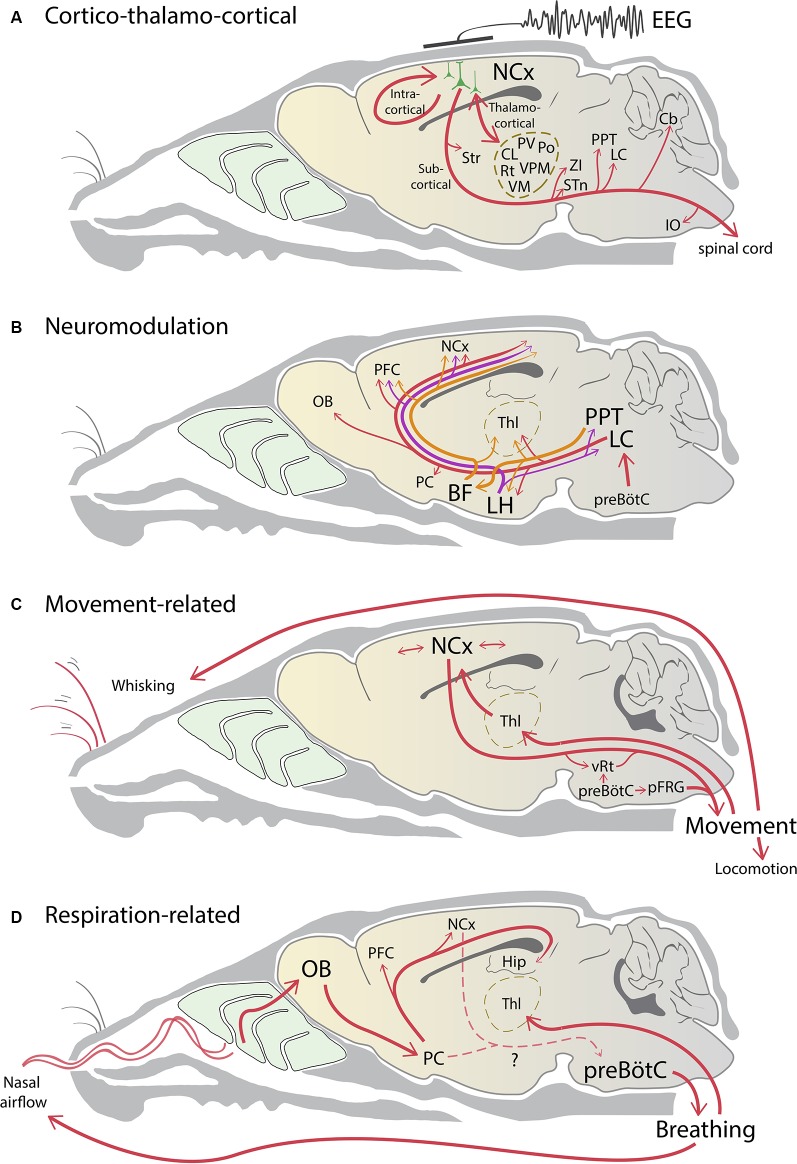
Neural pathways influencing brain state changes. **(A)** Rhythmic excitatory activity reverberates within local and intracortical circuits in the neocortex (NCx). The aggregate cortical activity collaborates with thalamic activity to form a thalamocortical loop. The resultant spiking activity in phase with brain state is broadcasted to the rest of the brain *via* many subcortical projections of layer 5 pyramidal neurons. **(B)** Noradrenergic (red), cholinergic (orange), and orexinergic (purple) neuromodulatory pathways project widely across the brain to influence brain state changes. Respiratory activity in the preBötzinger complex (preBötC) is coupled to the noradrenergic system to influence arousal and wakefulness. **(C)** Neocortical circuits and brainstem central pattern generators drive movement which can change brain state. Movement itself generates sensorimotor information that sends feedback to the cortex *via* the thalamus and contributes to cortico-thalamocortical activity. **(D)** preBötC sets the rhythm of nasal airflow, which in turn entrains respiration-related slow oscillatory activity *via* the olfactory system. This activity propagates across the neocortex and contributes to rhythmic cortical activity. Respiration can be under either voluntary or involuntary control, however, the pathways linking the neocortex and the piriform cortex (PC) to the preBötC are unclear (dashed lines). Note that for simplicity arrows do not indicate direct connectivity, and the line thickness does not indicate connection strength. BF, basal forebrain; Cb, cerebellum; Hip, hippocampus; CL, central lateral thalamus; IO, inferior olive; LC, locus coeruleus; LH, lateral hypothalamus; OB, olfactory bulb; PFC, prefrontal cortex; pFRG, parafacial respiratory group; Po, posterior medial thalamic nucleus; PPT, pedunculopontine nucleus; PV, paraventricular thalamic nucleus; Rt, reticular thalamic nucleus; STn, subthalamic nucleus, Str, striatum; Thl, thalamus; VM, ventromedial thalamic nucleus; VPM, ventroposterior medial thalamic nucleus; vRT, vibrissal reticular formation; ZI, zona incerta.

The elaborate long-range connectivity of layer 5 pyramidal neurons means that when cortical activity does change, activity in many subcortical areas follows the cortical rhythm. This effect of cortical activity is especially easy to monitor during slow-wave sleep, when activity spanning the entire cortical mantle fluctuates, and when sensory input and movement is minimal. When cortical activity fluctuates, the subcortical activity also shows phasic slow rhythmic activity in most targets of cortical axons (direct and multisynaptic) including thalamus (Steriade et al., 1993a,b; Contreras and Steriade, 1995; Contreras et al., 1996; Sherman and Guillery, 2011); striatum (Cowan and Wilson, 1994; Wilson and Kawaguchi, 1996); zona incerta (Barthó et al., 2007); subthalamus (Magill et al., 2004); globus pallidus (Magill et al., 2000; Tseng et al., 2001; Kasanetz et al., 2002); inferior olive (Rowland et al., 2010); locus coeruleus (Sara and Hervé-Minvielle, 1995); cholinergic pedunculopontine neurons (Roš et al., 2010; Motelow et al., 2015; Petzold et al., 2015); cerebellum and deep cerebellar nuclei (Ros et al., 2009; Rowland et al., 2010; Figure 1A). Most targets of pyramidal neurons show activity that is correlated with cortical slow waves, suggesting that in the quietly sitting animal, when input from the sensory world is negligible, these axons can drive changes in membrane potential, local field potentials, and even action potentials.

The thalamus is intimately connected to the cortex (Figure 1A): it is central to the thalamocortical loop, gates sensory input, and as mentioned above its activity fluctuates with cortical activity. However, the thalamus is not a unitary entity. For areas like the somatosensory cortex, multiple thalamic nuclei can affect cortical activity, including the ventroposterior medial (VPM), the posterior medial nucleus (POm), and the midline intra-laminar thalamic nuclei (Sherman and Guillery, 2011; Saalmann, 2014). Neurons in these nuclei also receive cortical input and project back to the cortex. The close bidirectional connection with the cortex and the heterogeneity of the thalamus suggests that at least some thalamic inputs could trigger fast changes in the cortical state (Steriade, 2006)—and so argue against a cortico-centric view. In contrast to the original studies that ablated the thalamus (Gloor et al., 1977; Steriade et al., 1993c; Timofeev et al., 2000), subsequent loss-of-function studies have shown that thalamic inactivation (i.e., pharmacologically or lesioning inputs to the thalamus) can deactivate the persistent depolarization in the cortex and enhance slow cortical rhythms (Aguilar et al., 2010; Poulet et al., 2012; Zagha et al., 2013). Furthermore, whisker stimuli working through the thalamus or direct activation of the thalamus can trigger changes in cortical state (Petersen et al., 2003; Sachdev et al., 2004; Hasenstaub et al., 2007; Poulet et al., 2012; Poulet and Crochet, 2019).

The work mentioned above does not fully capture the role of the thalamus in modulating, triggering or maintaining changes in the brain-state. A substantial amount of recent evidence implicates a variety of midline thalamic nuclei in changing brain states globally, primarily in awakening the brain. Activating cholinergic input to reticular thalamus, which inhibits the reticular neurons, promotes slow-wave sleep (Ni et al., 2016), but remarkably direct inhibition of the reticular thalamus does not have the same effect. Neurons in the paraventricular thalamus increase their firing before mice wake up and optogenetic inhibition of these neurons lowers epochs of wakefulness. These effects of the paraventricular thalamic neurons are mediated by the nucleus accumbens pathway, not the prefrontal cortex pathway (Ren et al., 2018). The ventromedial thalamus, in particular the calbindin-positive layer 1 projecting matrix neurons, increase their firing before behavioral arousal and before changes in the EEG. The activity of these neurons can trigger arousal from slow-wave sleep or anesthesia (Honjoh et al., 2018). In the whisker system, POm and VPM neurons increase firing during active states, and firing specifically in the VPM neurons (compared with POm) is more correlated with whisking behavior (Urbain et al., 2015). Recently, similar findings have been extended to sleeping mice, where spikes of VPM neurons are correlated with a stronger impact on the cortical LFP than spikes of POm neurons during NREM sleep (Urbain et al., 2019). In non-human primates, stimulation of the central lateral thalamus, a higher-order thalamic nucleus, has recently been shown to drive lamina specific (in infragranular cortical layers) changes in cortical states (Redinbaugh et al., 2020).

Taken together, these heterogenous thalamic nuclei have a role in changing cortical activity. However, for this activity to be manifested as a change in brain state the corticothalamic loop must be engaged to broadcast any effect globally to the rest of the brain (Aru et al., 2019). These effects of the thalamus on arousal, on changing cortical state also has to occur in concert with inputs from the hypothalamus (hypocretin/orexinergic) and from the locus coeruleus (noradrenergic), both of which can trigger arousal (Adamantidis et al., 2007; Carter et al., 2010; Eban-Rothschild et al., 2018).

## Neuromodulation and the Hypothalamus

By the middle of the last century, it was increasingly clear that structures in the brainstem have a substantial role in the modulation of cortical states (Jones, 2008, 2020). Early experiments of Moruzzi and Magoun (1949) showed that electrical stimulation of the brainstem i.e., the reticular activating system, and other subcortical structures could abolish cortical synchrony in the EEG, replacing it with low amplitude fast activity. Lesioning some of the same regions abolished fast EEG activity (Lindsley et al., 1949). We now understand that many of the deep structures in the brainstem, midbrain, and hypothalamus are part of a large, interconnected neuromodulatory network that acts both directly and indirectly on the cortex to change brain states (Figure 1B). Collectively, these structures, which include the locus coeruleus, pedunculopontine nucleus, tuberomammillary nucleus, and others constitute the reticular activating system (reviewed in Jones, 2008, 2020).

Each structure is associated with a particular neuromodulatory transmitter, and stimulation of each of these structures can induce cortical state changes. The ascending cholinergic projections from the brainstem, for example, when stimulated optogenetically, produce powerful changes in brain state, increasing gamma and reducing slow wave rhythms in the cortex; and optogenetic stimulation of the locus coeruleus causes transitions from sleep to wake (Carter et al., 2010; Scammell et al., 2017; Cissé et al., 2018). Many of these subcortical and brainstem nuclei contain diverse neuronal types and transmit multiple transmitters, and therefore the roles that each structure play are still being updated. For example, the basal forebrain, known for its cholinergic neurons, also contains cortical projecting GABAergic neurons that powerfully affect brain states and initiate cortical gamma-band oscillations (Anaclet et al., 2015; Kim et al., 2015; Xu et al., 2015). In the locus coeruleus phasic but not tonic activation differentially encodes saliency in the cortex (Vazey et al., 2018), further highlighting the complexity of these neuromodulatory systems.

A more recent addition to the neuromodulatory subcortical network is the orexin/hypocretin neurons, which were discovered in 1998 by two groups at the same time (Sakurai et al., 1998; de Lecea et al., 1998). Orexin/hypocretin neurons reside in the lateral hypothalamus and, like other neuromodulatory structures, send projections far and wide across the brain, including to most of the other neuromodulatory centers in the brain, with the densest innervation to the locus coeruleus, which strongly promotes wakefulness (Peyron et al., 1998; Scammell et al., 2017).

Many recent studies suggest that the orexin/hypocretin system functions to coordinate and potentiate other neuromodulatory systems that stabilize arousal (Alexandre et al., 2013). Orexin/hypocretin neurons were found to be the underlying cause of the sleep/wake disorder, narcolepsy (Chemelli et al., 1999; Peyron et al., 2000; Thannickal et al., 2000), strongly implicating orexin/hypocretin neurons in cortical state changes and arousal (de Lecea, 2015). Optogenetic and chemogenetic stimulation of these neurons promotes the transition to wakefulness (Adamantidis et al., 2007; Sasaki et al., 2011), whereas their inhibition promotes sleep (Tsunematsu et al., 2013). Orexin/hypocretin neurons also innervate the thalamus and neocortex. But unlike other cortical projecting neuromodulatory centers that diffusely innervate and affect a wide range of neurons across many layers of the neocortex, orexin/hypocretin neurons appear to exclusively target deep layer 6 (layer 6b). Application of orexin/hypocretin to cortical brain slices specifically depolarizes these deep-layer 6b neurons (Bayer et al., 2004; Wenger Combremont et al., 2016).

Although the indirect but powerful effects of orexin/hypocretin on arousal are clear, the direct pathway linking orexinergic activation to cortical activity has not been studied. Since deep-layer 6 neurons are strongly and specifically activated by orexin/hypocretin, these cortical neurons might help induce the arousal effects of orexinergic neurons. Deep-layer 6 neurons were recently shown to project to cortical and thalamic regions commonly associated with cortical arousal, including neocortical layer 5 and the non-specific thalamic nuclei (Viswanathan et al., 2017; Hoerder-Suabedissen et al., 2018). However, unlike layer 6a which is a key component of the cortico-thalamocortical loop, the deeper sublayer 6b is best driven by cortico-cortical projection pathways, suggesting two separate pathways that interact differently with the thalamus (Zolnik et al., 2020). These neurons are genetically diverse and the exact relationship between orexin/hypocretin sensitivity and their projections is not yet worked out. Nevertheless, it is interesting to note that many of the thalamic structures that deep layer 6 targets are also orexin/hypocretin sensitive (Bayer et al., 2002). For example, the thalamic neurons are targets of deep layer 6 and as noted above is a powerful driver and regulator of cortical state; and many of the other subcortical neuromodulatory centers, including the pedunculopontine nucleus, project to non-specific thalamic nuclei and exert their influence on cortex by thalamocortical projections. The role of the lateral hypothalamus in stabilizing cortical arousal might be assisted by deep layer 6 neurons that act both on the ascending thalamocortical projections and directly on layer 5 cortical pyramidal neurons that have numerous long-range intracortical targets.

Orexin/hypocretin neurons also make descending projections and target several key structures in the brainstem. Among these is the respiratory rhythm generator, pre-Bötzinger nucleus in the medulla, which contains neurons that are excited by orexin/hypocretin application (Young et al., 2005). Breathing is an active mechanism, that is in part modulated by the action of hypothalamic neurons. Breathing is often correlated with brain state (see below). Injection of orexin/hypocretin into the pre-Bötzinger nucleus increases inspiratory tidal volume, and knock-out of orexin/hypocretin receptor gene in mice reduces the CO_2_ induced increases in breathing and increases the occurrence of sleep apnea (Young et al., 2005). Taken together, these findings show that neuromodulatory circuits can interdependently promote cortical state changes in a complex and diverse manner.

## Behavior, Active Sensation and Brain States

“The behaving organism embedded in a particular environment is what generates feelings” (Koch, 2004, p. 9). In the “enactive or sensorimotor” account of consciousness, the body (i.e., behavior) is closely linked to conscious perception. While this link is not explicit and can be easily argued against (see Koch, 2004), the link between movement and changes in brain state is clear. In quietly sitting, but awake mice, cortical activity is often dominated by low frequency 1–5 Hz (similar to respiratory rhythm, see below), fluctuations in the membrane potential (Wilson and Groves, 1981; Petersen et al., 2003; Ferezou et al., 2007), or fluctuations in multiunit activity (Ros et al., 2009). Intracranial EEG from human beings show similar low-frequency oscillations (Sachdev et al., 2015).

Movement (e.g., whisking or locomotion) abolishes the low frequencies and puts cortical circuits into a depolarized, persistently activated high gamma state (Petersen et al., 2003; Poulet et al., 2012; Polack et al., 2013; Zagha et al., 2013; Urbain et al., 2015). In the whisker system, even after the sensory nerve is cut, whisking initiates state changes (Poulet and Petersen, 2008; Poulet et al., 2012).

Even though movement can change brain state, two additional consequences of movement should be noted. One is that movement activates a variety of cortical circuits, and as mentioned above, the cortical pyramidal neuron links many sensorimotor brain systems together (Figure 1C). Thus, the interaction between these cortical circuits alone, without overt movement or the participation of the thalamus, can change brain state (Zagha et al., 2013). Second, movement is related to the phase and frequency of respiration. The latter point is important to consider because movement—at least orofacial movements in rats and mice—are modulated by respiration. Respiration can serve as a “common clock” for aligning diverse processes and behaviors (Cao et al., 2012; Moore et al., 2013; Ranade et al., 2013; Kleinfeld et al., 2014; McElvain et al., 2018).

While each breath does not reach conscious perception, it can be brought under voluntary control (Davenport et al., 2007). In this instance, sensorimotor processing is contingent upon a shift from autonomous respiratory rhythm to active sniffing bouts (from 1–5 Hz basal rate to 5–14 Hz in rodents). Active sensing by sniffing is coordinated with attentional processes and rhythmic movements (e.g., head direction or fine orofacial movements like whisking) that enable selective sampling routines (Schroeder et al., 2010; Moore et al., 2013; Jordan et al., 2018). This view seems paradigmatic in rodents as they are macrosmatic animals that sniff to sample their environment. Humans on the other hand favor vision and audition as a major source of information about the outside world and are less reliant on sniffing. However, studies in human beings have shown that cognitive processing of non-olfactory tasks is closely tied to the respiratory cycle (Nakamura et al., 2018; Perl et al., 2019), and that artificial puffs of air directed at the nares can change the subjective sensation of an altered state of consciousness (Piarulli et al., 2018).

Global brain oscillations, coupled to breathing, are coming into light as a general neural principle that may provide a configuration for temporally organizing brain activity across distant brain regions. Often these respiration-related brain rhythms are discussed in the context of the olfactory system—that they are driven by entrainment to nasal airflow. Though this is perfectly reasonable, as discussed below, there could also be other pathways that carry respiration-related information to link breathing behavior to brain-state.

## Respiration Affects Rhythmic Brain Activity

*Lord Adrian* was the first to report that “Normal breathing (without odor stimulation) produces a regular series of large potential waves in the pyriform area at each inspiration” (Adrian, 1942, p. 472). Years later, Fontanini et al. (2003) demonstrated that slow oscillations (<1.5 Hz) in the olfactory bulb and piriform cortex correlated with respiration in anesthetized rats (Fontanini and Bower, 2006). Subsequent studies in anesthetized and awake rodents have shown that respiratory rhythms modulate the oscillations (<5 Hz during anesthesia or quiet awake; >5 Hz during active sniffing) in brain regions downstream to the olfactory system (e.g., orbitofrontal cortex, the prelimbic cortex, and the hippocampus; Yanovsky et al., 2014; Lockmann et al., 2016; Biskamp et al., 2017; Liu et al., 2017; Kszeghy et al., 2018; Moberly et al., 2018; Rojas-Líbano et al., 2018), and also structures that are not associated with the olfactory system (e.g., somatosensory, primary motor, and primary visual cortices; Ito et al., 2014; Rojas-Líbano et al., 2018; Tort et al., 2018b). The respiration-related oscillations selectively modulate the 80–120 Hz gamma frequency band, the power of which attenuates as it propagates away from the olfactory bulb to cortical structures (Zhong et al., 2017). These studies have been extended to the human brain showing that respiratory rhythms are locked to nasal, but not oral inhalation, suggesting that respiration modulates brain activity even though humans rely less on olfactory information compared with rodents (Zelano et al., 2016; Herrero et al., 2018; Perl et al., 2019). The respiration-related modulation of low frequency and gamma-band oscillations are often overlooked because respiration frequency overlaps with both low frequencies (1–4 Hz) and with theta frequency bands (Tort et al., 2018a). When respiration is monitored, it is possible to differentiate between the respiration and theta coupling of spiking activity of cortical and hippocampal neurons (Ito et al., 2014; Yanovsky et al., 2014; Nguyen Chi et al., 2016; Biskamp et al., 2017; Zhong et al., 2017). Together, these findings suggest that olfaction-related respiratory rhythms are present globally across the brain, that is, respiration modulates rhythmic activity in multiple cortical areas.

One pathway that can generate respiration-related rhythms involves sensory signals generated in response to external nasal airflow that propagates to the cortex *via* olfactory pathways (Figure 1D) and then reverberates within the recurrent connections of the cortical network itself (Bagur and Benchenane, 2018; Tort et al., 2018a). The nasal airflow drawn by breathing is necessary for these rhythms because these oscillations disappear once the nasal airway is bypassed using tracheotomy (Fontanini et al., 2003; Phillips et al., 2012; Ito et al., 2014; Yanovsky et al., 2014; Lockmann et al., 2016), and can be reinstated with rhythmic artificial nasal air puffs (Phillips et al., 2012; Lockmann et al., 2016). Furthermore, eliminating bulbar activity, surgically or chemically, strongly reduces respiration-related oscillations in brain regions downstream to the olfactory bulb while sparing other frequency bands (i.e., theta, gamma; Phillips et al., 2012; Biskamp et al., 2017; Liu et al., 2017; Tantirigama et al., 2017).

A different pathway that is often overlooked in the discussion of respiratory-related rhythms originates from the brainstem. The act of respiration itself generates sensory signals not only from the nasal epithelium as discussed earlier, but also from mechanoreceptors in the chest, skin, and muscles that are continually moved by respiration. Brain stem nuclei that manage breathing connect with the thalamus, where thalamic neurons fire in synchrony with respiration (Chen et al., 1992; Pattinson et al., 2009; Yang and Feldman, 2018). From the thalamus, these respiration-related signals echo to many brain regions that are involved in respiratory proprioception and the qualitative evaluation of respiration (e.g., diencephalon, limbic structures, and neocortex; Chen et al., 1992; Davenport and Vovk, 2009). Recently, a loss-of-function study showed that silencing nucleus reuniens of the thalamus reduces the 2–5 Hz coherence between the prefrontal cortex and hippocampus without significantly affecting coherence for theta oscillation (Roy et al., 2017). Additionally, recent causal evidence shows that a set of neurons in the Pre-Botzinger complex can influence the brain-state. Manipulation of these neurons does not affect resting respiratory activity, however, it can dramatically change arousal or vigilance *via* direct connections to noradrenergic neurons in the locus coeruleus (Yackle et al., 2017). As mentioned earlier, orexin/hypocretin neurons also target the pre-Bötzinger complex (Young et al., 2005), and are likely to coordinate with the noradrenergic system in the locus coeruleus. Given that the brainstem circuits managing respiration are ultimately setting the rhythm of respiration (Ramirez and Baertsch, 2018), and thus the rhythm of chest movement and entry of air into the nares, these circuits are likely to play a role in modulating brain state in collaboration with neuromodulatory pathways.

## The Cortical Pyramidal Neuron—The Key to Understanding Brain State Transitions

Here, we have tackled the various cortical/subcortical interactions that combine to influence brain rhythms (Figure 1). The thalamocortical loops, neuromodulatory signals, activity generated by movement and respiration all interact with each other in a manner that is not yet completely understood. However, their effect to produce a change in brain state is ultimately manifested across the pyramidal neuron population. The resultant signal associated with the system-wide firing of various interacting neurons and brain areas results in specific changes in the flow of extracellular cortical currents, which make up the bulk of the signal finally referred to as “brain state” (or EEG). This raises three interesting points that revolve around cortical pyramidal neurons.

The first thing to consider is that pyramidal neurons are not “point neurons” but rather large cells that span several cortical layers (Larkum et al., 2018). This means that the influence of inputs arriving at different lamina will engage different compartments of pyramidal neurons that depend exquisitely on both the precise locations of the input on the dendritic tree and on the intrinsic properties of the neuron. These synaptic interactions can be quite baroque under certain spatiotemporal input distributions (Larkum, 2013; Stuart and Spruston, 2015). A well-studied example of this is the generation of NMDA spikes and calcium spikes in the distal dendrites, which can couple with input to the soma and trigger a burst output (Palmer et al., 2012, 2014; Stuart and Spruston, 2015). Importantly, this input/output transformation enabled by calcium spikes is strongly correlated to brain state (Phillips et al., 2016; Suzuki and Larkum, 2017; Seibt et al., 2017), and recent work shows that when the brain-state changes, the coupling between the soma and dendrite also changes (Suzuki and Larkum, 2020). This means that the influence of input from subcortical structures on the cortex must be gauged in the light of its specific effect on the active dendritic properties of pyramidal neurons, and how they can contribute to producing changes in the brain-state (Phillips et al., 2016; Aru et al., 2019).

Second point is that the output of pyramidal neurons and their subcortical targets interact in a complex way. In particular, the intrinsic properties of pyramidal neurons lead them to switch their firing mode (e.g., from sparse to regular firing, or bursting) as a function of the spatiotemporal pattern of inputs, which in turn is presumed to have a variable influence on their target neurons as a function of the short-term synaptic plasticity at their output synapses (Williams, 2005). To reiterate, during state transitions, it is these neurons that can modify and modulate the activity of many subcortical targets that are involved in brain-state, including the thalamus, brainstem/hypothalamic neuromodulatory systems, motor systems and respiratory systems.

Third, as discussed above, many long-range influences on brain-state are neuromodulatory in nature (e.g., cholinergic, orexinergic, noradrenergic, etc.), and arrive into specific layers of the cortex (Jacob and Nienborg, 2018). This means that at any moment a stereotypical, spatiotemporal pattern of activity onto a pyramidal neuron can be modified under the influence of neuromodulation. We are still only at the beginning of understanding how neuromodulation can modify the somatodendritic properties of pyramidal neurons and influence the brain-state on a moment-to-moment basis (e.g., Williams and Fletcher, 2019; Suzuki and Larkum, 2020).

All in all, there is a level of complexity in understanding and explaining the precise rhythms encompassed by brain-states that are rarely addressed in descriptions of system-wide interactions usually involving simple “block diagrams.” We predict that the key to understanding brain state transitions will require experiments and models that include these intricacies about pyramidal neurons.

## Conclusion

Our understanding of how brain state changes, and the circuits involved in producing these changes is rapidly expanding. Many circuits related to changes in brain state—i.e., the pathways through the thalamus, neuromodulatory systems in the hypothalamus and brainstem, and the link between breathing and orofacial behaviors, among them—have been revealed. Although each of these pathways is important for changing brain-state, perhaps each one alone is insufficient to trigger brain state changes, unless they collaborate to produce a system-wide change. The structural and functional interactions between them are still not clear, and our current challenge is to understand how these diverse set of cellular properties, circuits, and pathways interact to modify brain state at different timescales.

## Author Contributions

MT, TZ, BJ, ML, and RS wrote the manuscript.

## Conflict of Interest

The authors declare that the research was conducted in the absence of any commercial or financial relationships that could be construed as a potential conflict of interest.
